# Respiratory Sequelae of Prematurity in School-Age Children: Is Bronchopulmonary Dysplasia Still the Primary Risk Factor?

**DOI:** 10.3390/children13010005

**Published:** 2025-12-19

**Authors:** Milena Bjelica, Gordana Vilotijević Dautović, Slobodan Spasojević, Tanja Radovanović, Milica Plazačić

**Affiliations:** 1 Institute for Child and Youth Health Care of Vojvodina, Hajduk Veljkova 10, 21000 Novi Sad, Serbia; gordana.vilotijevic-dautovic@mf.uns.ac.rs (G.V.D.); slobodan.spasojevic@mf.uns.ac.rs (S.S.); rtanja0@gmail.com (T.R.); milica.plazacic@mf.uns.ac.rs (M.P.); 2 Faculty of Medicine, University of Novi Sad, Hajduk Veljkova 3, 21000 Novi Sad, Serbia

**Keywords:** premature birth, morbidity, respiratory tract diseases, lung function, child

## Abstract

**Background/Objectives**: Respiratory morbidity in preterm infants has been widely studied, with evidence showing that individuals born prematurely experience more frequent respiratory symptoms, airflow obstruction, and radiological lung abnormalities throughout life. **Methods**: This study included 150 children aged 6 to 11 years, divided into two groups. The preterm group (*n* = 90) consisted of children born before 32 weeks of gestation, while the control group (*n* = 60) included term-born children. All participants underwent spirometry and completed a respiratory health questionnaire. **Results**: A significantly higher proportion of preterm children exhibited respiratory morbidity compared to term-born peers (χ^2^ = 7.035; *p* = 0.030). However, no significant differences were found between preterm children with and without bronchopulmonary dysplasia (BPD) defined at day 28 (BPD28) (χ^2^ = 0.002; *p* = 0.968) or BPD defined at 36 weeks postmenstrual age (BPD36) (χ^2^ = 0.029; *p* = 0.864). Lung function parameters—forced vital capacity (FVC), forced expiratory volume in 1 s (FEV_1_), FEV_1_/IVC, maximal expiratory flow at 25%, 50% and 75% of FVC (MEF_25_, MEF_50_, and MEF_75_) were significantly lower in the preterm group (*p* = 0.004 for FVC and *p* < 0.001 for all other parameters). No significant differences were observed between BPD and non-BPD subgroups in any lung function parameter. BPD36 was found to be a stronger predictor of respiratory morbidity (OR = 1.214) than BPD28 (OR = 1.093), although neither BPD28 nor BPD36 were statistically significant predictors. **Conclusions**: This study underscores the long-term respiratory consequences of prematurity and challenges the traditional view of BPD as the primary determinant of poor respiratory outcomes. Our findings suggest that prematurity itself, rather than BPD, may play a more central role.

## 1. Introduction

Respiratory morbidity in preterm infants, from infancy through to adulthood, has been the subject of numerous studies over the past few decades, especially as an increasing number of preterm infants survive and grow into adulthood. These studies generally indicate that individuals born prematurely experience more frequent respiratory symptoms, partially reversible airflow obstruction, and pulmonary radiological abnormalities during both childhood and adulthood.

Preterm patients are at greater risk of developing chronic lung disease, as they often do not reach their optimal peak lung function in early adulthood. There is also a potential—though not yet sufficiently substantiated—hypothesis of an accelerated decline in lung function, particularly, if these individuals are exposed to environmental noxious agents. This is precisely why pediatric and adult pulmonologists are now encountering a completely new population of patients with specific respiratory pathologies, while questions regarding the monitoring and treatment of these patients remain unresolved [1,2,3].

Preterm children, especially those born before 32 weeks of gestation and those with bronchopulmonary dysplasia (BPD), are at higher risk of respiratory infections, wheezing episodes, and hospitalizations due to respiratory deterioration in the first years of life compared to term-born children. This risk is most pronounced in the first two years of life and gradually decreases by the age of five, although it remains higher than expected for the patient’s age [4,5,6]. Additionally, preterm birth is often recognized as a risk factor for the development of asthma, and some of the potential reasons associated with prematurity include immaturity of the immune system, structural and functional changes in the pulmonary parenchyma, and frequent respiratory infections [7,8]. However, respiratory symptoms associated with preterm birth or BPD are often unjustifiably attributed to asthma [2].

Since it was first described in 1967, the characteristics of BPD have significantly evolved due to the introduction of new therapeutic strategies, leading to corresponding changes in its definition. Over time, multiple definitions of BPD have been proposed, reflecting shifts in the at-risk population and clinical presentation, as well as the ongoing need to more accurately describe disease severity and predict long-term outcomes. While all definitions are based on the requirement for oxygen therapy or respiratory support, they differ in the postnatal time point at which BPD is diagnosed—ranging from the 28th day of life to 36 weeks postmenstrual age (PMA) [9,10,11,12,13]. Some definitions also incorporate severity stratification [11,12,13]. Additionally, different definitions are used across various centers, making the monitoring and study of this disease even more complex. The influence of BPD on the respiratory health of children from school age onward remains unclear, particularly in light of evolving neonatal care and changes in the clinical characteristics of BPD. This manuscript addresses this topic with the aim of clarifying the extent to which prematurity itself and BPD, as an independent risk factor, contribute to long-term respiratory outcomes. It also assesses the predictive value of current BPD definitions for respiratory morbidity in school-aged children. By doing so, the study aims to bridge the gap between early-life diagnoses of prematurity and BPD and respiratory health in later childhood, thereby guiding long-term follow-up strategies for these patients.

## 2. Materials and Methods

### 2.1. Study Design and Setting

The study was conducted as a prospective, observational, single-center study at the Pediatric Clinic of the Institute for Child and Youth Health Care of Vojvodina in Novi Sad. The research included a total of 150 participants divided into two groups.

### 2.2. Participants (Inclusion and Exclusion Criteria)

The first group consisted of 90 participants aged 6 to 11 years who were born preterm (before completing 32 weeks of gestation). Following a review of the perinatal medical histories of preterm infants, a cohort of patients born before 32 weeks of gestational age was identified and subsequently contacted by telephone. The study included only those patients whose parents agreed to bring their child for an outpatient pulmonology evaluation as part of the research protocol. By reviewing medical records from the neonatal period, it was determined whether the participants had BPD according to two different definitions (at day 28 (BPD28)) and at 36 weeks postmenstrual age (PMA) (BPD36)). Exclusion criteria for this group included: congenital lung malformations and other congenital or chronic lung diseases (except asthma), severe congenital heart defects, severe psychomotor developmental delay, neuromuscular diseases, and acute respiratory illness. The second group consisted of 60 participants aged 6 to 11 years who were born at term (at or after 37 weeks of gestation). The control group was composed of children hospitalized for diseases of other organ systems, excluding respiratory conditions. Exclusion criteria for this group included: congenital lung malformations and other congenital or chronic lung diseases (including asthma), severe congenital heart defects, severe psychomotor developmental delay, neuromuscular diseases, and acute respiratory illness.

Since asthma was not an exclusion criterion in the preterm group (to better assess the overall respiratory morbidity in these participants) and considering that asthma could influence lung function and respiratory morbidity, a separate subgroup was formed excluding participants currently receiving asthma treatment (*n* = 9). Analyses and comparisons were also conducted specifically for this subgroup. The structure of preterm participants is summarized in Table 1.

### 2.3. Procedures

The study included lung function testing using spirometry (PowerCube Body+ (GANSHORN Medizin Electronic GmbH, Niederlauer, Germany; SCHILLER AG, Baar, Switzerland)) for all participants, following the guidelines of the American Thoracic Society and the European Respiratory Society. The following spirometry parameters were analyzed: inspiratory vital capacity (IVC), forced vital capacity (FVC), forced expiratory volume in 1 s (FEV_1_), ratio of FEV_1_ to inspiratory vital capacity (FEV_1_/IVC), maximal expiratory flow at 25% of FVC (MEF_25)_, maximal expiratory flow at 50% of FVC (MEF_50_), and maximal expiratory flow at 75% of FVC (MEF_75_). Body plethysmography was performed in 76 preterm subjects and 40 term-born subjects who demonstrated adequate cooperation, using the same device. The following parameters were analyzed: residual volume (RV), total lung capacity (TLC), and the RV/TLC ratio. In addition, total airway resistance (Rawtot) was measured.

All participants underwent a parent-completed questionnaire designed for the study to collect anamnestic data on respiratory diseases and symptoms. To assess respiratory morbidity in both preterm and term-born participants, we assessed four parameters in the past 12 months: wheezing episodes (defined as the use of inhaled bronchodilators), pneumonia, dry nighttime cough, and symptoms during physical activity. The presence of any of the four parameters was interpreted as the presence of respiratory morbidity, while the absence of all four was interpreted as the absence of respiratory morbidity. A comparison was made between preterm and term-born participants regarding respiratory morbidity, and two BPD definitions (BPD28 and BPD36) were validated and compared in terms of their predictive value for respiratory morbidity in school-aged children.

### 2.4. Ethics

The study was approved by the Ethics Committee of the Institute for Child and Youth Health Care of Vojvodina (approval number 3774-1, dated 29 September 2021). Prior to inclusion in the study, each parent or legal guardian signed an informed consent form.

### 2.5. Statistical Analysis

For statistical data analysis, the software package Statistical Package for Social Sciences (SPSS, version 21) was used. Numerical variables were presented as means (arithmetic mean) and measures of variability (range, standard deviation), while categorical variables were expressed as frequencies and percentages. Comparisons of numerical variables between two groups were performed using the Student’s t-test or the non-parametric Mann–Whitney U test. For comparisons involving three or more groups, one-way analysis of variance (ANOVA) or the non-parametric Kruskal–Wallis test was applied. Differences in the distribution of categorical variables were assessed using the chi-square (χ^2^) test. To examine associations between two or more variables and to generate appropriate statistical models, univariate and multivariate binary logistic regression analyses were conducted. A *p*-value of less than 0.05 was considered statistically significant.

## 3. Results

### 3.1. BPD Assessment in Patients

The study included a total of 150 participants aged between 6 and 11 years. Out of the total number of participants, 90 were born before 32 completed weeks of gestation, while 60 were born at term (≥37 weeks of gestation). The average age of the participants was 8.7 years. Based on the definition involving oxygen dependency on the 28th postnatal day, 59 participants were diagnosed with BPD, whereas according to the definition at 36 weeks of PMA, 30 participants were diagnosed with BPD. Further classification of participants was conducted based on the severity of BPD using two different definitions. According to the National Institute of Child Health and Human Development—NICHD criteria, Jobe et al. [11], 31 participants (34.4%) had no BPD, 29 (32.2%) had mild BPD, 26 (28.9%) had moderate BPD, and 4 (4.4%) had severe BPD. Additionally, using the classification published by Jensen et al. [13], 60 participants (66.7%) were classified as having no BPD, 26 (28.9%) as grade 1, 3 (3.3%) as grade 2, and 1 (1.1%) as grade 3.

### 3.2. Respiratory Morbidity

A comparison of respiratory morbidity parameters in school-aged children was conducted among three groups of participants (preterm children with and without BPD28/BPD36 and term-born children). A statistically significantly higher percentage of preterm participants, both with and without BPD28, had respiratory morbidity compared to term-born children (χ^2^ = 7.035; *p* = 0.030). No statistically significant difference was found between preterm participants with and without BPD28 (χ^2^ = 0.002; *p* = 0.968). Similarly, a significantly higher percentage of preterm participants, both with and without BPD36, had respiratory morbidity compared to term-born children (χ^2^ = 7.070; *p* = 0.029). No statistically significant difference was found between preterm participants with and without BPD36 (χ^2^ = 0.029; *p* = 0.864) (Table 2).

When patients receiving asthma therapy were excluded, the previously observed statistically significant difference in respiratory morbidity between preterm participants (with and without BPD28) and term-born children was no longer present (χ^2^ = 2.380; *p* = 0.304), as well as between those with and without BPD36 and term-born children (χ^2^ = 4.188; *p* = 0.123) as presented in Table 3.

The predictive strength of the two BPD definitions (BPD28 and BPD36) for respiratory morbidity was assessed using logistic regression analysis. The analysis was adjusted for sex and age of the participants and is presented in Table 4. Neither BPD28 nor BPD36 were statistically significant predictors, and there was no statistically significant difference between two definitions (*p* = 0.89). When using the variable “preterm/term-born” as a predictor in univariate logistic regression analysis, it was found that preterm-born participants had nearly 3.8 times (OR = 3.776) higher odds of having respiratory morbidity compared to term-born participants as presented in Table 4. Logistic regression analysis was also used to assess the statistical significance and predictive strength of respiratory morbidity for BPD definitions based on severity as presented in Table 5. It was found that neither of these definitions was a statistically significant predictor of respiratory morbidity. However, moderate/severe BPD had higher OR, while mild BPD was not associated with increased risk (OR = 0.99). According to the definition by Jensen et al. [13], BPD grade 2/3 had higher OR than grade 1, but again without the statistically significant difference between these BPD grades (*p* = 0.85).

### 3.3. Lung Function

Concerning spirometry parameters, preterm participants had statistically significantly lower values of FVC, FEV_1_, FEV_1_/IVC, MEF_25_, MEF_50_, and MEF_75_ compared to term-born participants. With respect to body plethysmography parameters, preterm infants demonstrated statistically significantly higher values of RV/TLC ratio. No statistically significant differences were observed in the remaining parameters. These results are presented in Table 6. Comparison of lung function parameters among participants with and without BPD28/BPD36 is shown in Table 7. No statistically significant differences were found between the groups with and without BPD28/BPD36 in any parameter. These findings remained unchanged even after excluding participants currently receiving asthma therapy.

## 4. Discussion

### 4.1. Respiratory Morbidity

In our study, we compared parameters of respiratory morbidity in school-aged children across three groups of participants (preterm children with and without BPD28/BPD36 and term-born children). A statistically significantly higher percentage of preterm-born participants had respiratory morbidity compared to those born at term, while no statistically significant differences were observed between participants with and without BPD according to either definition. However, when participants with asthma were excluded from the preterm group, the statistically significant difference disappeared. Similar results were reported by Cazzato et al., who in their study found no difference in respiratory morbidity over the past two years in school-aged children between those who had BPD and those who did not [14]. However, a significantly larger number of studies indicate that respiratory symptoms are more frequent in preterm children compared to their term-born peers, with greater use of respiratory-related therapies and a higher prevalence of asthma, especially in the group of patients who had BPD [15,16,17].

To date, numerous definitions of BPD have been published. New definitions aim to better predict long-term respiratory morbidity, which is essential for providing parents with adequate prognostic information, developing protocols for long-term follow-up, ensuring appropriate funding and supporting research in new preventive and therapeutic strategies. Long-term respiratory morbidity in children who had BPD shows variable results in the literature, indicating limitations of current BPD definitions and the potential influence of other factors on respiratory health [18]. So far, studies have examined the predictive power of BPD definitions in relation to respiratory morbidity in the first years of life. For example, Shennan et al. concluded that the need for oxygen therapy up to day 28 is not a good predictor of respiratory morbidity by age two, especially in infants born before 30 weeks of gestation (positive predictive value (PPV) was 38%). On the other hand, oxygen dependency at 36 weeks postmenstrual age was found to be a good predictor of respiratory morbidity regardless of gestational age (PPV was 63%) [10].

To our best knowledge there are no studies in the current literature that examine the ability of BPD definitions to predict respiratory morbidity in school-aged children. Since it is known that BPD can have consequences on respiratory health much later in life, validating the definitions of this disease in that context is extremely important. In our study, two definitions of BPD (BPD28 and BPD36) were evaluated as predictors of respiratory morbidity in school-aged children, and neither proved to be statistically significant predictor. Similarly, BPD definitions that take severity into account were also not identified as statistically significant predictors of respiratory morbidity. These findings suggest that while BPD definitions may contribute to risk assessment, they should be interpreted within a broader clinical context that includes additional perinatal and postnatal factors. On the other hand, preterm birth significantly predicted respiratory morbidity, with preterm children (with or without BPD) having 3.8 times higher odds (OR = 3.776) than term-born peers. These findings are consistent with the literature, which suggests that the presence of BPD is not an adequate marker for long-term respiratory morbidity. Preterm birth is a distinct pathophysiological process that requires repeated evaluation over time. Therefore, BPD, which is defined at a specific point in time, is an inadequate parameter for assessing long-term respiratory outcomes [19,20]. Our study results indicate that the presence of BPD, especially in its severe forms, may influence the presence of respiratory morbidity, but this influence is not statistically significant—unlike preterm birth. On the other hand, the BPD36 definition may better reflect the long-term consequences of this disease on an individual’s respiratory health compared to the BPD28 definition. This could be explained by the fact that children with BPD36 are typically diagnosed at a later postnatal age and require prolonged oxygen therapy compared to those with BPD28, highlighting the potential influence of more advanced disease forms. The lack of statistical significance may reflect sample size limitations or other confounding factors. These findings underscore the importance of cautious interpretation and suggest that further studies with larger cohorts are needed to clarify the predictive value of BPD definitions. Moreover, in the context of long-term prediction of respiratory morbidity, and in light of ongoing advances in neonatal care, future revisions of BPD definitions may increasingly aim to capture the most severe and persistent forms of chronic lung injury.

### 4.2. Lung Function

Prematurity is associated with reduced pulmonary function parameters, most notably in patients with lower gestational age and those with BPD. This deficit deepens during childhood, with a loss of FEV_1_ of 0.1 Z-score per year, increasing the risk of developing chronic obstructive pulmonary disease. Since children born preterm often fail to reach the expected peak in lung function during growing up and may potentially experience an accelerated decline in lung function, they are at risk of entering adulthood with lung function below the expected level for their age. Additional negative effects may come from genetic predisposition, exposure to tobacco smoke, respiratory infections, and gene–environment interactions during critical phases of lung development [21].

Monitoring lung function in preterm children is one of the key principles in the early detection of chronic respiratory morbidity [20,21]. Although most studies indicate that preterm children have poorer lung function than term-born children, the results in the literature are quite heterogeneous, especially regarding BPD [22]. It should also be noted that lung function data are obtained from participants capable of cooperating adequately during testing. Children with neurocognitive deficits are generally excluded from studies, even though they represent a group whose vital functions, including lung function, are most compromised by prematurity [23].

Preterm children, especially those with BPD, typically exhibit obstructive ventilatory disorders with reduced FEV_1_ values, which persist into adulthood [24,25,26,27,28,29]. A meta-analysis by Kotecha et al., which included participants aged 5–23 years, showed that preterm children without BPD had 7.2% lower FEV_1_ values, those with BPD28 had 16.2% lower values, and those with BPD36 had 18.9% lower values compared to term-born children [30]. In our study, preterm participants had statistically significantly lower values of FVC, FEV_1_, FEV_1_/IVC, MEF_25_, MEF_50_, and MEF_75_ compared to term-born children. Specifically, preterm children had 6.6% lower FVC, 11.7% lower FEV_1_, 9.2% lower FEV_1_/IVC, 21.4% lower MEF_25_, 16% lower MEF_50_, and 9.4% lower MEF_75_. The difference in IVC was 3.5% in favor of term-born participants, but without statistical significance. However, no statistically significant differences were found between groups with and without BPD28 or BPD36 in any parameter, although most of these parameters were lower in children with BPD. Similar results were reported by other authors. Verheggen et al., in a study of 118 children born before 32 weeks of gestation aged 4–8 years, found that all spirometry parameters were lower in preterm participants, but no differences were found between those with and without BPD [31]. Cazzato et al., in children born before 32 weeks of gestation and aged 8–9 years, found statistically lower FVC, FEV_1_, and FEF_25–75_ values in the preterm group, but again no difference was observed regarding BPD [14]. Three additional studies compared preterm participants with and without BPD and consistently reported no differences in spirometry parameters [32,33,34]. In a study of prematurely born adolescents, Doyle et al. evaluated the impact of two BPD definitions on lung function. They reported that children with BPD had significantly lower lung function parameters, but the choice of definition did not alter the statistical differences observed between those with and without BPD [35]. Hagman et al. reported that, at 12 years of age, children born very preterm with a previous diagnosis of BPD exhibited greater airflow obstruction (lower FEV_1_/FVC and absolute value of FEF_25-75_), increased peripheral airway resistance, and reduced diffusion capacity compared to preterm peers without BPD. However, no differences were observed in FEV_1_, FVC, or static lung volumes [36]. To better determine which perinatal factors influence lung function in school-aged preterm children, Hart et al. assessed the impact of perinatal factors on lung function in 544 preterm children (<34 weeks of gestation) aged 7–12 years and found that gestational age and intrauterine growth restriction negatively affected lung function, while BPD did not, concluding that BPD is not an optimal marker of future lung function [37]. Our study supports this, showing that birth before 32 weeks of gestation is a significant risk factor for both impaired lung function and increased respiratory morbidity, while the impact of BPD, regardless of its definition, was not confirmed. On the other hand, a significant number of studies highlight BPD as an additional factor negatively affecting lung function in school-aged children, with these children having lower spirometry parameters compared to preterm children without BPD [3,17,38,39,40,41,42,43]. However, a meta-analysis by Kotecha et al., which included 86 studies, showed that FEV_1_ values in preterm patients with BPD increased as year of birth increased, while these values remain unchanged in preterm children without BPD. These results are explained by the introduction of surfactant therapy and other advances in neonatal care [44]. The study conducted by Bårdsen et al. further supports this observation, demonstrating that lung function deficits following extremely preterm birth compared with term-born individuals decreased progressively with each decade of birth between 1980 and 2000 [24]. Therefore, it is important to consider the birth year of preterm children when comparing results, and to keep in mind that the most accurate comparisons can be made when the birth years in two studies are similar. On the other hand, this may be one of the reasons why BPD is no longer a dominant risk factor for reduced lung function and why statistical significance is lacking when comparing lung function between patients with and without BPD, as was the case in our study.

Since asthma can affect lung function in preterm children, our study also analyzed spirometry parameters after excluding participants currently receiving asthma therapy from the preterm group. The results remained unchanged. This excluded the potential influence of asthma on the obtained results.

This study has several important limitations. The relatively small sample size restricted the robustness of the analyses, as following preterm children into school age and obtaining their consent to participate proved challenging. Furthermore, the exclusion of children with psychomotor developmental delays may have introduced bias, since this subgroup often experiences the highest rates of respiratory morbidity, thereby reducing the generalizability of the findings. Another limitation was the small number of patients with severe BPD, which is closely related to the exclusion of children with neurodevelopmental impairments, as severe BPD frequently co-occurs with such impairments. As a result, the most vulnerable subgroup may have been underrepresented, which could have contributed to the absence of statistically significant differences in respiratory morbidity and lung function outcomes between the BPD and non-BPD groups.

## 5. Conclusions

This study highlights the long-term respiratory consequences of premature birth and offers a new perspective on BPD, which was for years considered as the main risk factor for increased respiratory morbidity and impaired lung function. In this study, we did not demonstrate an effect of BPD independent of prematurity. Instead, we showed that prematurity itself is associated with reduced pulmonary function parameters, while BPD does not appear to influence these outcomes. Although prematurity was linked to higher respiratory morbidity compared with term-born children, this association disappeared after excluding patients with asthma. This suggests that the observed problems may be asthma-related, or that prematurely born children presenting with symptoms could be misdiagnosed with asthma—a concern already raised in the literature. Furthermore, neither of the current definitions of BPD proved to be strong predictors of respiratory morbidity in school-aged children. Taken together, these results underscore the need for further research into the long-term respiratory sequelae of prematurity, particularly as the nature and extent of these outcomes are likely to evolve alongside ongoing advances in neonatal care.

## Figures and Tables

**Table 1:** Structure of preterm participants in numbers.

Included	Excluded	Participants with Asthma	Participants with BPD28	Participants with BPD36
90	235	9	59	30

**Table 2:** Comparison of respiratory morbidity between preterm-born school-aged children with/without BPD28/BPD36 and term-born children.

Respiratory Morbidity	No BPD28		BPD28		No BPD36		BPD36		Term-Born		Preterm/ Term	No BPD28/BPD28	No BPD36/ BPD36
	N	%	N	%	N	%	N	%	N	%	*p*, χ^2^	*p*, χ^2^	*p*, χ^2^
Yes	8	25.8%	15	25.4%	15	25.0%	8	26.7%	5	8.3%	0.03, 7.035	0.968, 0.002	0.864, 0.029
No	23	74.2%	44	74.6%	45	75.0%	22	73.3%	55	91.7%			

BPD28—diagnosis of BPD made at day 28, BPD36—diagnosis of BPD made at 36 weeks postmenstrual age, N—number, *p*—probability value, χ^2^—chi-square test.

**Table 3:** Comparison of respiratory morbidity between preterm-born school-aged children with/without BPD28/BPD36 and without asthma and term-born children.

Respiratory Morbidity	No BPD28 and no Asthma		BPD28, No Asthma		No BPD36 and No Asthma		BPD36, No Asthma		Term-Born		Preterm Without Asthma (with + Without BPD28)/Term	Preterm Without Asthma (with + Without BPD36)/Term
	N	%	N	%	N	%	N	%	N	%	*p*, χ^2^	*p*, χ^2^
Yes	5	17.9%	9	17.0%	7	13.5%	7	26.7%	5	8.3%	0.304, 2.380	0.123, 4.188
No	23	82.1%	44	83.0%	45	86.5%	22	73.3%	55	91.7%		

BPD28—diagnosis of BPD made at day 28, BPD36—diagnosis of BPD made at 36 weeks postmenstrual age, N—number, *p*—probability value, χ^2^—chi-square test.

**Table 4:** Prediction of respiratory morbidity in preterm-born school-aged children using two definitions of BPD and preterm birth.

		*p*	OR	95% CI
BPD28	No	0.865	1.00 ^a^	0.393–3.043
	Yes		1.093	
BPD36	No	0.711	1.00 ^a^	0.435–3.393
	Yes		1.214	
Preterm birth Term birth		0.012	1.00 ^a^ 3.776	1.347–10.585

BPD28—diagnosis of BPD made at day 28, BPD36—diagnosis of BPD made at 36 weeks postmenstrual age, OR—odds ratio, CI—confidence interval, *p*—probability value, ^a^—reference value; logistic regression.

**Table 5:** Respiratory morbidity prediction based on BPD definitions.

		*p*	OR	95% Cl
JOBE, NICHD	No BPD	0.985	1.00 ^a^	
	Mild		0.989	0.299–3.264
	Moderate/Severe	0.754	1.208	0.371–3.930
JENSEN	No BPD	0.764	1.00 ^a^	
	Grade 1		1.180	0.405–3.436
	Grade 2/3	0.733	1.530	0.133–17.656

BPD—bronchopulmonary dysplasia, JOBE, NICHD—BPD definition based on severity by Jobe et al. [11], JENSEN—BPD definition based on severity by Jensen et al. [13], OR—odds ratio, CI—confidence interval, *p*—probability value, ^a^—reference value; logistic regression.

**Table 6:** Comparative analysis of lung function parameters in preterm versus term-born participants.

		N	Mean	SD	Min	Max	*p*
IVC (%)	Preterm	90	86.8933	15.71128	52.00	135.00	0.153 ***
	Term	60	90.3917	15.56801	58.00	162.00	
FVC (%)	Preterm	90	82.5556	13.53739	55.00	130.00	0.004 ***
	Term	60	89.1950	14.94917	63.00	167.70	
FEV_1_ (%)	Preterm	90	89.5022	14.73814	49.00	132.00	<0.001 ***
	Term	60	101.2067	13.90687	77.00	170.50	
FEV_1_/IVC	Preterm	90	84.8523	13.87573	56.00	113.00	<0.001 ***
	Term	60	94.0832	10.52135	73.00	125.00	
MEF_25_ (%)	Preterm	90	81.9444	28.08429	27.00	165.00	<0.001 **
	Term	60	103.3750	24.89065	46.00	185.00	
MEF_50_ (%)	Preterm	90	77.2722	21.74148	29.00	126.00	<0.001 ***
	Term	60	93.2633	14.05078	61.00	128.00	
MEF_75_ (%)	Preterm	90	77.0567	18.72633	21.00	126.00	<0.001 ***
	Term	60	86.4183	14.94205	51.00	114.30	
Raw_tot_ (kPa·s/L)	Preterm Term	76 40	0.5141 0.5098	0.26485 0.19361	0.10 0.24	1.56 0.87	0.819 ***
TLC (%)	Preterm Term	76 40	111.657 109.4250	16.86460 16.17197	73.00 81.00	172.00 154.00	0.493 **
RV (%)	Preterm Term	76 40	180.6447 157.6500	59.91877 62.74063	50.00 35.00	329.00 324.00	0.056 **
RV/TLC (%)	Preterm Term	76 40	39.74 35.33	9.767 10.123	15 11	63 54	0.024 **

IVC—inspiratory vital capacity, FVC—forced vital capacity, FEV_1_—forced expiratory volume in 1 s, FEV1/IVC—ratio of FEV_1_ to inspiratory vital capacity, MEF_25_—maximal expiratory flow at 25% of FVC, MEF_50_—maximal expiratory flow at 50% of FVC, MEF_75_—maximal expiratory flow at 75% of FVC, RV—residual volume, TLC—total lung capacity, Raw_tot_—total airway resistance, N—number, SD—standard deviation, Min—minimum value, Max—maximum value, *p*—probability value; ** *t* test, *** Mann–Whitney test.

**Table 7:** Comparative analysis of lung function parameters among preterm children with and without BPD28/BPD36.

		N	Mean	SD	Min	Max	No BPD/BPD
							*p*
IVC (%)	No BPD28	31	89.2258	16.58254	64.00	135.00	0.379 **
	BPD28	59	85.6678	15.23520	52.00	134.00	
	No BPD36	60	89.2167	16.76567	60.00	135.00	0.079 **
	BPD36	30	82.2467	12.33629	52.00	111.00	
FVC (%)	No BPD28	31	81.6452	14.24909	55.00	130.00	0.508 **
	BPD28	59	83.0339	13.24825	55.00	110.00	
	No BPD36	60	83.1167	14.28534	55.00	130.00	0.598 **
	BPD36	30	81.4333	12.05357	57.00	107.00	
FEV_1_ (%)	No BPD28	31	90.3226	13.73168	49.00	132.00	0.809 **
	BPD28	59	89.0712	15.33721	63.00	119.00	
	No BPD36	60	90.5000	15.25990	49.00	132.00	0.282 **
	BPD36	30	87.5067	13.66311	65.00	118.00	
FEV_1_/IVC	No BPD28	31	82.2581	12.24464	57.00	107.00	0.477 **
	BPD28	59	86.2154	14.57413	56.00	113.00	
	No BPD36	60	83.0833	13.72156	56.00	111.00	0.181 **
	BPD36	30	88.3903	13.72618	60.00	113.00	
MEF_25_ (%)	No BPD28	31	84.5806	24.75045	33.00	165.00	1 ***
	BPD28	59	80.5593	29.79544	27.00	149.00	
	No BPD36	60	81.6500	27.21965	27.00	165.00	1 ***
	BPD36	30	82.5333	30.21045	38.00	143.00	
MEF_50_ (%)	No BPD28	31	80.6129	20.43637	29.00	119.00	0.384 **
	BPD28	59	75.5169	22.36549	31.00	126.00	
	No BPD36	60	77.9333	21.89874	29.00	126.00	0.801 **
	BPD36	30	75.9500	21.73284	31.00	110.00	
MEF_75_ (%)	No BPD28	31	79.2903	18.93356	21.00	126.00	0.465 **
	BPD28	59	75.8831	18.67130	34.00	126.00	
	No BPD36	60	78.5667	18.83593	21.00	126.00	0.418 **
	BPD36	30	74.0367	18.44560	34.00	126.00	
Raw_tot_ (kPa·s/L)	No BPD28	26	0.4888	0.21612	0.10	0.86	0.917 **
	BPD28	50	0.5272	0.28814	0.17	1.56	
	No BPD36	51	0.5222	0.27059	0.10	1.56	0.547 **
	BPD36	25	0.4976	0.25738	0.19	1.11	
TLC (%)	No BPD28	26	113.1923	17.82362	86.00	172.00	1 ***
	BPD28	50	110.8600	16.47262	73.00	145.00	
	No BPD36	51	112.7647	16.03819	86.00	172.00	1 ***
	BPD36	25	109.4000	18.57418	73.00	145.00	
RV (%)	No BPD28	26	182.0769	59.84575	80.00	318.00	1 ***
	BPD28	50	179.9000	60.55028	50.00	329.00	
	No BPD36	51	179.1765	60.75054	55.00	329.00	1 ***
	BPD36	25	183.6400	59.30281	50.00	285.00	
RV/TLC (%)	No BPD28	26	39.38	10.108	21	63	1 ***
	BPD28	50	39.92	9.684	15	57	
	No BPD36	51	39.41	10.423	15	63	1 ***
	BPD36	25	40.40	8.431	16	54	

IVC—inspiratory vital capacity, FVC—forced vital capacity, FEV_1_—forced expiratory volume in 1 s, FEV1/IVC—ratio of FEV_1_ to inspiratory vital capacity, MEF_25_—maximal expiratory flow at 25% of FVC, MEF_50_—maximal expiratory flow at 50% of FVC, MEF_75_—maximal expiratory flow at 75% of FVC, RV—residual volume, TLC—total lung capacity, Rawtot—total airway resistance, BPD—bronchopulmonary dysplasia, N—number, SD—standard deviation, Min—minimum value, Max—maximum value, *p*—probability value; ** Mann–Whitney test, *** Bonferroni post hoc test.

## Data Availability

The original contributions presented in this study are included in the article. Further inquiries can be directed at the corresponding author.

## Abbreviations

The following abbreviations are used in this manuscript:

BPD	bronchopulmonary dysplasia
BPD28	diagnosis of BPD made at day 28
BPD36	diagnosis of BPD made at 36 weeks postmenstrual age
PMA	postmenstrual age
IVC	inspiratory vital capacity
FVC	forced vital capacity
FEV_1_	forced expiratory volume in 1 s
FEV1/IVC	ratio of FEV_1_ to IVC
MEF_25_	maximal expiratory flow at 25% of FVC
MEF_50_	maximal expiratory flow at 50% of FVC
MEF_75_	maximal expiratory flow at 75% of FVC
RV	residual volume
TLC	total lung capacity
Rawtot	total airway resistance
